# The relationship of macular vessel density and foveal avascular zone with pulse wave velocity and central blood pressure in healthy adults: Persian cohort study

**DOI:** 10.1186/s40942-024-00616-7

**Published:** 2024-12-18

**Authors:** Hamid Reza Heidarzadeh, Elaheh Ebrahimi Miandehi, Nasser Shoeibi, Mohammad Reza Ansari Astaneh, Seyedeh Maryam Hosseini, Majid Abrishami, Mehrdad Motamed Shariati, Saeid Eslami, Parnian Arjmand, Mojtaba Abrishami

**Affiliations:** 1 Eye Research Center, Mashhad University of Medical Sciences, Qarani Blvd, Mashhad, 9195965919 Iran; 2 Persian Cohort Research Center, Mashhad University of Medical Sciences, Mashhad, Iran; 3 Department of Medical Informatics, Faculty of Medicine, Mashhad University of Medical Sciences, Mashhad, Iran; 4 Department of Medical Informatics, Amsterdam UMC, Amsterdam Public Health, University of Amsterdam, Amsterdam, The Netherlands; 5 Pharmaceutical Research Center, School of Pharmacy, Mashhad University of Medical Sciences, Mashhad, Iran; 6 Mississauga Retina Institute, Mississauga, ON Canada; 7 Ocular Oncology Service, Department of Ophthalmology and Visual Sciences, University of Toronto, Toronto, Canada

**Keywords:** Optical coherence tomography angiography (OCTA), Vessel density (VD), Foveal avascular zone (FAZ), Pulse wave velocity (PWV), Arterial age

## Abstract

**Background:**

To evaluate the correlation of macular vessel density (VD) and foveal avascular zone (FAZ) parameters measured on optical coherence tomography angiography (OCTA) with systemic arterial stiffness using pulse wave velocity (PWV), pulse wave analysis, arterial age, and central blood pressure (CBP) measurements in healthy subjects.

**Methods:**

In a comparative, cross-sectional, observational study, healthy adults who participated in the PERSIAN Cohort study at Mashhad University of Medical Sciences were included in this study. The study involved using a spectral domain OCTA device to obtain 3 × 3 and 6 × 6 mm scans of the macular superficial capillary plexus (SCP) VD, deep capillary plexus (DCP) VD, and FAZ vascular analysis. Additionally, we used the SphygmoCor XCEL System (AtCor, Itasca, IL) to analyze systemic vascular parameters like CBP and PWV.

**Results:**

The study included 296 healthy participants with a mean age of 39.2 ± 6.7 years, and 152 subjects (51.3%) were female. Female participants were found to have higher DCP VD and FAZ area values. Age showed a negative correlation with SCP and DCP VDs. PWV showed a negative correlation with parafoveal DCP VDs, but no correlation was observed between macular VDs and aortic diastolic and systolic pressures.

**Conclusion:**

In conclusion, age was found to have a negative impact on macular SCP and DCP VDs. In addition, higher arterial stiffness was found to correlate with a lower parafoveal DCP VD value. These findings suggest macular OCTA parameters may be used as early markers of systemic arterial disease.

**Supplementary Information:**

The online version contains supplementary material available at 10.1186/s40942-024-00616-7.

## Introduction

The retinal vascular flow is supplied through the retinal vasculature and the choriocapillaris [1]. The choriocapillaris is the main vascular supply to the outer retina and the only source of blood supply to the foveola. Within the macula, the inner retina is primarily supplied by the superficial and deep vascular plexuses (SVP and DVP, respectively), while the outer retina is supplied by outer retinal and choriocapillaris vasculature [2].

Pulse wave velocity (PWV) is an established method of measuring arterial stiffness, which is an indicator of vascular aging and a predictive marker of cardiovascular events [3, 4]. PWV can be measured on magnetic resonance imaging angiography or on ultrasound imaging [5]. Arterial stiffness affects vascular impedance, which relates to changes in arterial pressure and blood flow. PWV is dependent on the cardiac wall stiffness which is affected by age, systemic disease, and environmental factors [5]. PWV has been correlated to various eye diseases, including diabetic retinopathy, retinal vein occlusion, age-related macular degeneration, and glaucoma [6–13].

Optical coherence tomography angiography (OCTA) is a non-invasive imaging modality of the retinal microcirculation, aiding in the diagnosis and monitoring of retinal disease. Systemic diseases such as Alzheimer’s, diabetes, and more recently, corona virus disease 2019 (COVID-19), have been shown to impact the microvasculature changes measured through OCTA studies [14–22].

Vascular density (VD) and the foveal avascular zone (FAZ) are crucial markers for evaluating retinal microvasculature, offering valuable insights into retinal health and potential pathologies. VD, measured using OCTA, quantifies the proportion of blood vessel flow within the retina [14]. Variations in this density are linked to several retinal conditions, such as diabetic retinopathy, age-related macular degeneration, and retinal vein occlusion. These variations can signal compromised retinal perfusion, which, if left unaddressed, may lead to visual impairment [14, 23, 24].

The FAZ, located at the center of the retina and lacking in blood vessels, is crucial for maintaining high visual acuity. Changes in the size and shape of the FAZ have been linked to retinal diseases, where an enlarged or irregular FAZ often corresponds to visual dysfunction [25, 26]. Recent studies underscore the significance of these parameters, demonstrating strong associations between vascular density, FAZ alterations, and disease severity in conditions such as diabetic retinopathy [27]. The introduction of OCTA has allowed for early detection of these microvascular changes, making it an invaluable tool in diagnosing and monitoring retinal diseases.

The current literature on the correlation between PWV and macular VDs, or the FAZ, is limited, with most studies focusing on control groups or small-scale investigations. While these studies offer some insight, they often lack the depth and scope to understand the intricate relationship between systemic vascular health fully, as PWV indicates, and retinal microvascular parameters such as macular VDs and FAZ. Our study aims to address this gap by providing a more comprehensive analysis of this relationship in our cohort study on subjects with no recognized chronic disease. We explore how PWV, a marker of arterial stiffness and cardiovascular risk, correlates with macular VDs and FAZ changes across a broader and more diverse population. By doing so, we hope to contribute valuable data to the field and offer new perspectives on the potential interplay between systemic vascular conditions and retinal health.

## Methods

### Study participants

All healthy adults participating in the PERSIAN Organizational Cohort Study (POCM) conducted at the Imam Reza Hospital in Mashhad, Iran, which is affiliated with Mashhad University of Medical Sciences (MUMS) were included. Past ocular and medical historical data as well as demographic data including age and gender were collected from all subjects. Height, weight, body mass index (BMI), blood pressure, and resting heart rate measurements were collected under standard conditions.

Exclusion criteria were as follows: chronic systemic disease, active pregnancy or breastfeeding, a prior or current diagnosis of glaucoma or ocular hypertension, clinically apparent retinal disease, or a history of refractive or intraocular surgery, absolute spherical equivalent refractive error of greater than five diopters, cylindrical myopic refractive error of more than six diopters, and hyperopic refractive error of more than four diopters. We also excluded patients with chronic use of any prescribed or non-prescribed drugs such as oral contraceptive. An internal medicine specialist reviewed all patients’ laboratory results in the cohort data, and none had significant abnormalities that required intervention.

Participants with ocular media opacity that hindered high-quality imaging or a reduced OCTA scan quality (i.e., quality scan index less than 8/10) were also excluded from the analysis. Refractive error was assessed using a KR-1 Auto Kerato-Refractometer (Topcon Medical Systems, Inc., Tokyo, Japan).

### Imaging technique

**Fig. 1:**
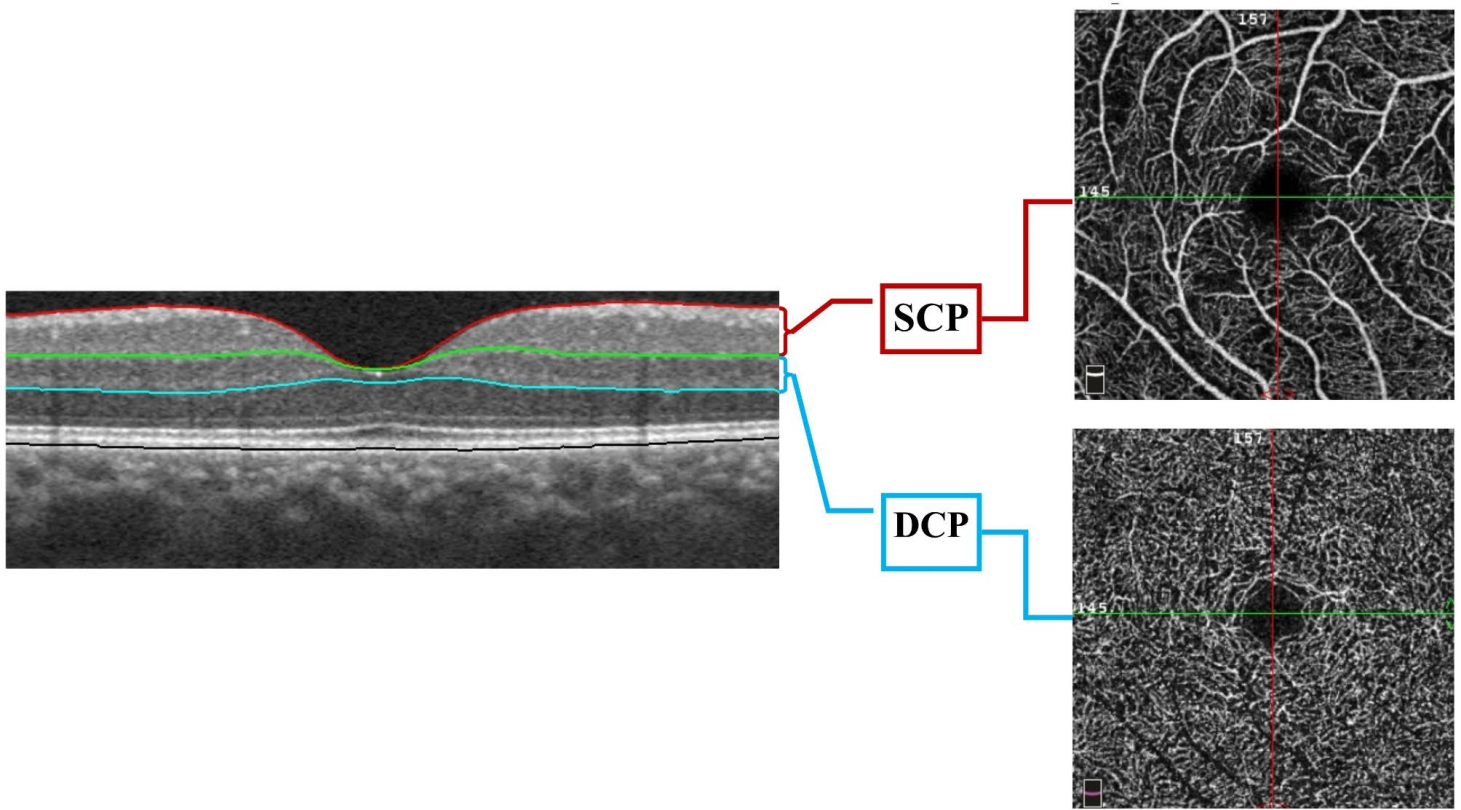
Optical coherence tomography angiography (OCTA) image shows segmentation slabs on B-Scan and corresponding enface inner and outer retinal angiograms. Superficial capillary plexus (SCP) is between the inner limiting membrane and the outer boundary of the inner plexiform layer. Deep capillary plexus (DCP) is between the outer boundary of the inner plexiform layer and the outer boundary of the outer plexiform layer

The AngioVue (RTVue XR Avanti, Optovue, Fremont, California, USA; Software Version 2018.0.0.14) OCTA system was imaged at an A-scan rate of 70,000 scans per second, using a light source of 840 nm and a 45 nm bandwidth. All OCT B-scans were replicated to avoid discrepancies. We conducted macular cube scans in horizontal and vertical directions using the AngioRetina 3 × 3 and 6 × 6 mm scans protocol with AngioVue 3D Projection Artifact Removal. Automated default segmentation was used for measurements with preset SCP and DCP settings (Fig. 1).

The study analyzed only the data from the right eye VD values. Only high-quality images centered on the fovea with a quality scan index of at least 8/10 were included. Retina specialists (MA, SMH, MA, and NS) checked all images for segmentation errors and all images with motion artefact were excluded.

**Fig. 2:**
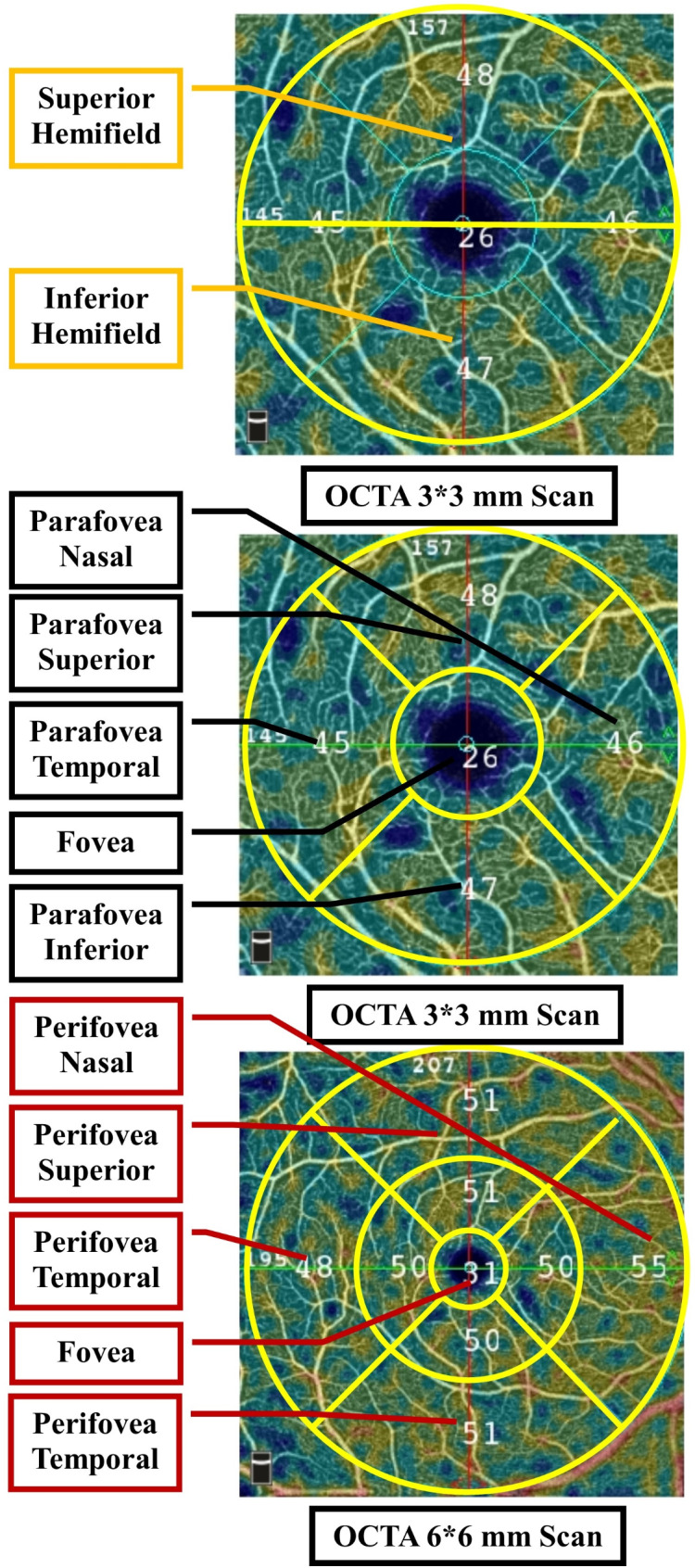
Macular optical coherence tomography angiography (OCTA) images showing vessel density measurement maps in superficial inner retina enface OCT images in 3*3 and 6*6 mm protocols

We measured various features from the AngioAnalytic report, including FAZ surface area, perimeter size, acircularity index (AI), foveal VD-300 (FD-300) area density, FD-300 length density, as well as VD of the macula at the SCP and DCP levels. The fovea was marked as the central 1 mm circle in the macula, with parafovea and perifovea being defined as rings around the fovea with 3 and 6 mm diameters, respectively. We used the 3 × 3 mm scans to extrapolate VD and FAZ data for the fovea and parafovea. Additionally, we utilized the 6 × 6 mm scans to evaluate the VD in the perifoveal region (Fig. 2). We used VD data from the right eye to analyze differences and correlations among participants [28].

### Pulse wave velocity

The SphygmoCor XCEL System (AtCor Medical Inc., Itasca, IL) was used to analyze central blood pressure (CBP) and PWV. As part of the test protocol, participants were required to fast for at least 6 h and refrain from tobacco, alcohol, or caffeine for at least 12 h prior to the test.

The test was non-invasive and involved two steps after the participant rested in a supine position for 10–15 min. CBP measurements were taken by recording cuff pulsations during three consecutive inflation-deflation cycles of the central pressure waveform at the brachial artery. PWV was measured using an ultrasound tonometer on the carotid pulse and a cuff around the thigh. Augmentation index (AIx) was also measured, representing the percentage of central pulse pressure due to a late systolic peak. The SphygmoCor reference age values were also extracted from the device. This value estimates arterial age based on central pressure parameters in healthy individuals.

During the capture process, the waveform’s color changes based on the recording quality. Initially white, the waveform transitions to green when the capture conforms to the quality criteria and incorporates the minimum required number of quality pulses. Participants with unreliable PWV measurements were excluded.

### Statistical analysis

We examined variable distribution using the Shapiro-Wilk test and variance normality plots and checked for homogeneity using Levene’s test. We used independent-sample t-test and one-way ANOVA for comparisons based on data distribution and type, with a *p*-value of < 0.05 indicating statistical significance. The Pearson correlation coefficient test measured the correlation between study parameters. SPSS program version 16 for Windows was used for all statistical analyses (IBM SPSS Statistics, IBM Corporation, Chicago, IL, USA).

### Ethical considerations

The study followed the principles of the Declaration of Helsinki. The Regional Committee on Medical Ethics at Mashhad University of Medical Sciences, Mashhad, Iran (IR.MUMS.MEDICAL.REC.1401.229) approved the ethical aspects of the study. All participants provided written informed consent.

## Results

This study recruited 296 healthy individuals with an equal gender distribution (51.3% female). The average age of this cohort was 39.2 ± 6.7 years. Table 1 shows the demographic and baseline clinical findings. Male subjects had significantly higher values for all FAZ and VD parameters (*P* < 0.001), while no significant difference was observed between males and females for age or BMI. In our study, 14 (4.72%) cases indicated a history of smoking. The average exposure to cigarette smoking was 9.16 ± 21.29 pack-years, with a median of 1.77 pack-years.

**Table 1:** Demographic, clinical, and biochemical characteristics of study participants. BP: blood pressure, CAI: central augmentation index, AIx: augmentation index, PWV: pulse wave velocity, FBS: fasting blood sugar, LDL: low-density lipoprotein, HDL: high-density lipoprotein

	Total (*n* = 296)	Male (*n* = 144)	Female (*n* = 152)	*P*-value
Age (Year)	39.2 ± 6.7	39.8 ± 7.9	38.9 ± 5.9	0.462
Height (cm)	167 ± 9.7	174.8 ± 6.1	159.5 ± 6	**< 0.001**
Weight (kg)	72.1 ± 12.4	79.6 ± 11.1	65 ± 9	**< 0.001**
Body Mass Index (kg/m^2^)	25.8 ± 3.2	26 ± 3.2	25.5 ± 3.1	0.154
Systolic BP (mmHg)	104.4 ± 10.7	109.2 ± 10.7	99.8 ± 8.8	**< 0.001**
Diastolic BP (mmHg)	67.3 ± 7.2	70.1 ± 7.2	64.4 ± 6.3	**< 0.001**
FBS (mg/dL)	93.5 ± 4.2	96.1 ± 5.1	92.7 ± 4.7	**0.01**
LDL (mg/dL)	92.4 ± 27.7	93.7 ± 27.7	90.7 ± 26.9	0.058
HDL (mg/dL)	61 ± 11.9	61 ± 11.9	61 ± 11.8	0.914
Triglyceride (mg/dL)	117.1 ± 24.9	131.1 ± 21.2	100.3 ± 52.9	**< 0.001**
Total Cholesterol (mg/dL)	176.5 ± 25.4	182.5 ± 24.2	171.4 ± 24.9	**< 0.001**

The average scan quality for images with 3 × 3 mm and 6 × 6 mm dimensions were 8.8 ± 0.6 and 8.2 ± 0.7, respectively. Table 2 summarizes the OCTA scan VD values for all participants based on gender on 3 × 3 mm and 6 × 6 mm scans. In 3 × 3 scans, female participants had a higher mean value of DCP VD in all areas except for the foveal DCP VD, while male participants had a higher mean foveal SCP VD. The FAZ area was larger in female participants (0.25 mm^2^ vs. 0.30 mm^2^, *p* < 0.001). In the 6 × 6 mm OCTA scans, only the SCP VD value was found to be higher in female participants. Meanwhile, the mean perifoveal SCP VD values in the temporal, superior, nasal, and inferior sectors were all significantly higher in females. There were no significant differences in mean perifoveal DCP VD values based on gender.

**Table 2:** The 3 × 3 mm and 6 × 6 mm OCTA scan values in males and females. OCTA: optical coherence tomography angiography, VD: vessel density, SCP: superficial capillary plexus, DCP: deep capillary plexus, FAZ: foveal avascular zone, AI: acircularity index, FD: foveal vessel density

OCTA Parameter		Total (*n* = 296)	Male (*n* = 144)	Female (*n* = 152)	*P*-value
**3 × 3 mm Scan**	Whole Image SCP VD (%)	46 ± 2.4	45.9 ± 2.5	46 ± 2.3	0.991
	Superior Hemifield SCP VD (%)	45.8 ± 2.4	45.7 ± 2.5	45.9 ± 2.5	0.926
	Inferior Hemifield SCP VD (%)	46.1 ± 2.4	46 ± 2.5	46.1 ± 2.3	0.721
	Fovea SCP VD (%)	17.1 ± 5.6	18.5 ± 5.5	15.7 ± 5.3	**< 0.001**
	Parafovea SCP VD (%)	48.9 ± 2.5	48.8 ± 2.6	49.1 ± 2.5	0.527
	Parafovea Superior Hemifield SCP VD (%)	48.8 ± 2.6	48.7 ± 2.7	48.9 ± 2.6	0.524
	Parafovea Inferior Hemifield SCP VD (%)	49.1 ± 2.6	48.9 ± 2.7	49.2 ± 2.5	0.564
	Parafovea Temporal SCP VD (%)	47.2 ± 2.6	47.4 ± 2.8	47.2 ± 2.5	0.770
	Parafovea Superior SCP VD (%)	50.2 ± 2.8	49.9 ± 2.8	50.4 ± 2.8	0.120
	Parafovea Nasal SCP VD (%)	48.2 ± 2.7	48.3 ± 2.6	48.1 ± 2.7	0.854
	Parafovea Inferior SCP VD (%)	50.2 ± 2.9	50 ± 2.9	50.5 ± 2.8	0.244
	Whole Image DCP VD (%)	50.2 ± 2.9	49.5 ± 3	50.8 ± 2.8	**0.001**
	Whole Image Superior Hemifield DCP VD (%)	50.1 ± 3	49.4 ± 3	50.6 ± 2.9	**0.001**
	Whole Image Inferior Hemifield DCP VD (%)	50.3 ± 3	49.6 ± 3.1	50.9 ± 2.8	**0.001**
	Fovea DCP VD (%)	32.7 ± 6.8	34.6 ± 6.3	31.1 ± 6.8	**< 0.001**
	Parafovea DCP VD (%)	52.3 ± 2.9	51.2 ± 2.9	53.2 ± 2.6	**< 0.001**
	Parafovea Superior Hemifield DCP VD (%)	52.3 ± 3	51.3 ± 3	53.2 ± 2.8	**< 0.001**
	Parafovea Inferior Hemifield DCP VD (%)	52.4 ± 3	51.2 ± 3.1	53.2 ± 2.7	**< 0.001**
	Parafovea Temporal DCP VD (%)	52.9 ± 2.8	51.8 ± 2.9	53.6 ± 2.6	**< 0.001**
	Parafovea Superior DCP VD (%)	51.8 ± 3.3	50.7 ± 3.3	52.7 ± 3	**< 0.001**
	Parafovea Nasal DCP VD (%)	52.9 ± 2.9	51.8 ± 2.9	53.7 ± 2.8	**< 0.001**
	Parafovea Inferior DCP VD (%)	51.9 ± 3.3	50.7 ± 3.5	52.8 ± 2.9	**< 0.001**
	FAZ Area (mm^2^)	0.28 ± 0.10	0.25 ± 0.08	0.3 ± 0.10	**< 0.001**
	FAZ Perimeter (mm)	2.1 ± 0.4	2 ± 0.4	2.2 ± 0.4	**< 0.001**
	AI	1.13 ± 0.03	1.13 ± 0.04	1.12 ± 0.03	0.565
	FD (%)	50.5 ± 3.1	50.4 ± 3.1	50.7 ± 3.1	0.280
**6 × 6 mm Scan**	Whole Image SCP VD (%)	49.2 ± 2.6	48.6 ± 2.6	49.7 ± 2.5	**0.004**
	Perifovea Temporal SCP VD (%)	46.3 ± 2.9	45.8 ± 2.9	46.7 ± 2.7	**0.033**
	Perifovea Superior SCP VD (%)	49.4 ± 3.3	48.9 ± 3.3	49.9 ± 3.3	**0.019**
	Perifovea Nasal SCP VD (%)	53.7 ± 2.8	53.2 ± 2.9	54.2 ± 2.6	**0.007**
	Perifovea Inferior SCP VD (%)	50.1 ± 3.2	49.5 ± 3.2	50.5 ± 3.1	**0.048**
	Whole Image DCP VD (%)	49.2 ± 0.2	49 ± 3.9	49.3 ± 4.1	0.543
	Perifovea Temporal DCP VD (%)	54.5 ± 3.6	54.1 ± 3.5	54.8 ± 3.7	0.211
	Perifovea Superior DCP VD (%)	49.1 ± 4.7	49.2 ± 4.6	49.1 ± 4.8	0.828
	Perifovea Nasal DCP VD (%)	47.8 ± 5.1	47.4 ± 5.1	48.1 ± 5.2	0.270
	Perifovea Inferior DCP VD (%)	50.6 ± 5.2	50.6 ± 5.1	50.5 ± 5.5	0.779

Table 3 Shows the measures of arterial stiffness and its correlation with gender. PWV was significantly higher among male patients (*p* < 0.001). Table 4 displays the correlation between macula VD values obtained from 3 × 3 mm OCTA scans and study parameters like age, CBP, and PWV. The average VDs of the whole image SCP and DCP were negatively correlated with age (*r*=-0.146, *p* = 0.012 and *r*=-0.135, *p* = 0.021). Furthermore, the mean DCP VD of the complete image was negatively correlated with systolic and diastolic BP. The PWV negatively correlated with DCP VD sectors in the parafovea. Additionally, the parafoveal DCP VD negatively correlated with age, diastolic and systolic BP, heart rate, aortic diastolic and systolic pressures, and PWV values. Among the FAZ values in table 4, only the mean acircularity index (AI) positively correlated with age (*r* = 0.147, *p* = 0.012)

**Table 3:** Arterial stiffness measures of study participants. AIx: augmentation index, PWV: pulse wave velocity

Arterial stiffness measures	Total (*n* = 296)	Male (*n* = 144)	Female (*n* = 152)	*P*-value
Heart Rate (bpm)	67.2 ± 8.7	64.8 ± 9.1	69.1 ± 8	**< 0.001**
Ejection Duration (msec)	336 ± 18.2	333 ± 17.8	338.3 ± 17.7	**0.025**
Aortic Systolic (mmHg)	103.1 ± 8.7	105.6 ± 9.2	101.2 ± 7.9	**< 0.001**
Aortic Diastolic (mmHg)	70.1 ± 7.6	71.9 ± 7.3	68.6 ± 7.4	**0.001**
Mean Pressure (mmHg)	83.1 ± 8	84.7 ± 8.1	82 ± 7.6	**0.011**
Aix (%)	24.8 ± 10.9	21.7 ± 11.2	28.5 ± 9.4	**< 0.001**
PWV (m/s)	5.9 ± 1	6.3 ± 1	5.5 ± 0.9	**< 0.001**
SphygmoCor Reference Age (Year)	44.5 ± 16.7	46.6 ± 18.0	42.4 ± 15.2	**< 0.001**

**Table 4:** Correlation of the Pulse wave velocity parameters and the Macular OCTA Imaging Parameters in 3×3 resolutions. **Correlation is significant at the 0.01 level (2-tailed). *Correlation is significant at the 0.05 level (2-tailed). OCTA: optical coherence tomography angiography, VD: vessel density, SCP: superficial capillary plexus, DCP: deep capillary plexus, FD: foveal vessel density, BP: blood pressure, PWV: pulse wave velocity, Ref Age: SphygmoCor reference age, FAZ: foveal avascular zone, AI: acircularity index

OCTA Parameter	Age	Diastolic BP	Systolic BP	Heart Rate	Aortic Diastolic	Aortic Systolic	PWV	Ref Age
Whole Image SCP VD (%)	r=-0.146* p = 0.012	r = 0.052 p = 0.376	r = 0.064 p = 0.272	r=-0.001 p = 0.983	r = 0.042 p = 0.498	r = 0.042 p = 0.498	r = 0.046 p = 0.428	r=-0.038, p = 0.505
Superior Hemifield SCP VD (%)	r=-0.149* p = 0.011	r = 0.047 p = 0.422	r = 0.064 p = 0.276	r = 0.042 p = 0.497	r = 0.047 p = 0.441	r = 0.047 p = 0.441	r = 0.023 p = 0.698	r=-0.061, p = 0.282
Inferior Hemifield SCP VD (%)	r=-0.124* p = 0.034	r = 0.06 p = 0.304	r = 0.067 p = 0.247	r=-0.035 p = 0.572	r = 0.046 p = 0.455	r = 0.046 p = 0.455	r = 0.074 p = 0.205	r=-0.036, p = 0.527
Fovea SCP VD (%)	r=-0.037 p = 0.531	r = 0.06 p = 0.3	r = 0.127* p = 0.029	r=-0.089 p = 0.149	r = 0.064 p = 0.301	r = 0.064 p = 0.301	r = 0.057 p = 0.329	r = 0.035, p = 0.535
Parafovea SCP VD (%)	r=-0.123* p = 0.035	r = 0.037 p = 0.527	r = 0.053 p = 0.364	r = 0.01 p = 0.87	r = 0.037 p = 0.544	r = 0.037 p = 0.544	r = 0.054 p = 0.351	r=-0.061, p = 0.283
Parafovea Superior Hemifield SCP VD (%)	r=-0.118* p = 0.043	r = 0.042 p = 0.467	r = 0.068 p = 0.241	r = 0.046 p = 0.452	r = 0.04 p = 0.512	r = 0.04	r = 0.04 p = 0.492	r=-0.056, p = 0.325
						p = 0.512		
Parafovea Inferior Hemifield SCP VD (%)	r=-0.120* p = 0.041	r = 0.029 p = 0.615	r = 0.034 p = 0.556	r=-0.026 p = 0.674	r = 0.032 p = 0.606	r = 0.032 p = 0.606	r = 0.064 p = 0.273	r=-0.062, p = 0.271
Parafovea Temporal SCP VD (%)	r=-0.127* p = 0.03	r = 0.059 p = 0.314	r = 0.102 p = 0.08	r = 0.016 p = 0.799	r = 0.075 p = 0.222	r = 0.075 p = 0.222	r = 0.09 p = 0.122	r=-0.028, p = 0.622
Parafovea Superior SCP VD (%)	r=-0.119* p = 0.042	r = 0.036 p = 0.534	r = 0.033 p = 0.569	r = 0.044 p = 0.48	r = 0.027 p = 0.665	r = 0.027 p = 0.665	r = 0.02 p = 0.733	r=-0.043, p = 0.447
Parafovea Nasal SCP VD (%)	r=-0.105 p = 0.072	r = 0.008 p = 0.886	r = 0.053 p = 0.363	r=-0.007 p = 0.91	r = 0.019 p = 0.753	r = 0.019 p = 0.753	r = 0.035 p = 0.546	r=-0.074, p = 0.193
Parafovea Inferior SCP VD (%)	r=-0.104 p = 0.077	r = 0.035 p = 0.547	r = 0.01 p = 0.865	r=-0.012 p = 0.852	r = 0.019 p = 0.761	r = 0.019 p = 0.761	r = 0.051 p = 0.378	r=-0.079, p = 0.16
Whole Image DCP VD (%)	r=-0.135* p = 0.021	r=-0.127* p = 0.028	r=-0.135* p = 0.02	r = 0.106 p = 0.085	r=-0.08 p = 0.191	r=-0.08 p = 0.191	r=-0.083 p = 0.154	r=-0.035, p = 0.531
Whole Image Superior Hemifield DCP VD (%)	r=-0.133* p = 0.023	r=-0.142* p = 0.015	r=-0.137* p = 0.018	r = 0.113 p = 0.067	r=-0.074 p = 0.232	r=-0.074 p = 0.232	r=-0.084 p = 0.151	r=-0.035, p = 0.536
Whole Image Inferior Hemifield DCP VD (%)	r=-0.132* p = 0.024	r=-0.108 p = 0.063	r=-0.128* p = 0.028	r = 0.097 p = 0.116	r=-0.085 p = 0.167	r=-0.085 p = 0.167	r=-0.081 p = 0.162	r=-0.035, p = 0.532
Fovea DCP VD (%)	r=-0.068 p = 0.25	r = 0.066 p = 0.255	r = 0.106 p = 0.068	r=-0.078 p = 0.205	r = 0.068 p = 0.266	r = 0.068 p = 0.266	r = 0.059 p = 0.311	r = 0.041, p = 0.465
Parafovea DCP VD (%)	r=-0.149* p = 0.011	r=-0.188** p = 0.001	r=-0.203** p < 0.001	r = 0.151* p = 0.014	r=-0.122* p = 0.047	r=-0.122* p = 0.047	r=-0.139* p = 0.017	r=-0.051, p = 0.364
Parafovea Superior Hemifield DCP VD (%)	r=-0.149* p = 0.011	r=-0.196** p = 0.001	r=-0.203** p < 0.001	r = 0.152* p = 0.013	r=-0.119 p = 0.053	r=-0.119 p = 0.053	r=-0.144* p = 0.013	r=-0.055, p = 0.332
Parafovea Inferior Hemifield DCP VD (%)	r=-0.141* p = 0.016	r=-0.170** p = 0.003	r=-0.192** p = 0.001	r = 0.143* p = 0.02	r=-0.119 p = 0.052	r=-0.119 p = 0.052	r=-0.127* p = 0.029	r=-0.045, p = 0.422
Parafovea Temporal DCP VD (%)	r=-0.154** p = 0.008	r=-0.181** p = 0.002	r=-0.195** p = 0.001	r = 0.118 p = 0.054	r=-0.143* p = 0.02	r=-0.143* p = 0.02	r=-0.121* p = 0.038	r=-0.058, p = 0.303
Parafovea Superior DCP VD (%)	r=-0.126* p = 0.032	r=-0.177** p = 0.002	r=-0.203** p < 0.001	r = 0.159** p = 0.01	r=-0.108 p = 0.08	r=-0.108 p = 0.08	r=-0.124* p = 0.033	r=-0.047, p = 0.407
Parafovea Nasal DCP VD (%)	r=-0.156** p = 0.008	r=-0.201** p < 0.001	r=-0.205** p < 0.001	r = 0.134*	r=-0.113 p = 0.067	r=-0.113 p = 0.067	r=-0.159** p = 0.006	r=-0.054, p = 0.338
				p = 0.029				
Parafovea Inferior DCP VD (%)	r=-0.129* p = 0.027	r=-0.155** p = 0.008	r=-0.166** p = 0.004	r = 0.158** p = 0.01	r=-0.102 p = 0.097	r=-0.102 p = 0.097	r=-0.121* p = 0.037	r=-0.039, p = 0.493
FAZ Area (mm2)	r = 0.008 p = 0.887	r=-0.077 p = 0.189	r=-0.096 p = 0.099	r = 0.07 p = 0.254	r=-0.056 p = 0.366	r=-0.073 p = 0.234	r=-0.052 p = 0.37	r=-0.035, p = 0.539
FAZ Perimeter (mm)	r = 0.027 p = 0.641	r=-0.068 p = 0.24	r=-0.092 p = 0.115	r = 0.075 p = 0.225	r=-0.03 p = 0.626	r=-0.05	r=-0.052 p = 0.377	r=-0.026, p = 0.649
						p = 0.42		
AI	r = 0.147* p = 0.012	r = 0.098 p = 0.094	r = 0.061 p = 0.298	r=-0.002 p = 0.973	r = 0.096 p = 0.119	r = 0.117 p = 0.058	r = 0.051 p = 0.378	r = .112*, p = 0.048
FD (%)	r=-0.097 p = 0.099	r=-0.077 p = 0.186	r=-0.00004 p = 0.999	r = 0.012 p = 0.852	r=-0.004 p = 0.949	r=-0.017 p = 0.78	r=-0.016 p = 0.79	r=-0.08, p = 0.157

The results of the 6 × 6 mm scans indicate a negative correlation between the mean whole image SCP VD and both age and PWV (*r*=-0.132, *p* = 0.023 and *r*=-0.132, *p* = 0.023, respectively). Conversely, no correlation was observed between OCTA perifovea VDs and other studied parameters (Table 5)

**Table 5:** Correlation of the pulse wave velocity parameters and the macular OCTA imaging parameters in 6×6 resolutions. **Correlation is significant at the 0.01 level (2-tailed). *Correlation is significant at the 0.05 level (2-tailed). OCTA: optical coherence tomography angiography, VD: vessel density, SCP: superficial capillary plexus, DCP: deep capillary plexus, BP: blood pressure, PWV: pulse wave velocity, Ref Age: SphygmoCor reference age

OCTA Parameter	Age	Diastolic BP	Systolic BP	Heart Rate	Aortic Diastolic	Aortic Systolic	PWV	Ref Age
Whole Image SCP VD (%)	*r*=-0.119* *p* = 0.042	*r*=-0.027 *p* = 0.64	*r*=-0.085 *p* = 0.143	*r* = 0.037 *p* = 0.552	*r*=-0.049, *p* = 0.425	*r*=-0.039 *p* = 0.523	*r*=-0.132* *p* = 0.023	*r*=-0.032, *p* = 0.578
Perifovea Temporal SCP VD (%)	*r*=-0.154** *p* = 0.008	*r* = 0.003 *p* = 0.954	*r*=-0.051 *p* = 0.384	*r* = 0.028 *p* = 0.65	*r*=-0.019, *p* = 0.758	*r*=-0.016 *p* = 0.79	*r*=-0.085 *p* = 0.146	*r*=-0.049, *p* = 0.389
Perifovea Superior SCP VD (%)	*r*=-0.121* *p* = 0.038	*r*=-0.002 *p* = 0.976	*r*=-0.072 *p* = 0.214	*r* = 0.046 *p* = 0.453	*r*=-0.039, *p* = 0.526	*r*=-0.015 *p* = 0.806	*r*=-0.168** *p* = 0.004	*r*=-0.001, *p* = 0.986
Perifovea Nasal SCP VD (%)	*r*=-0.116* *p* = 0.047	*r*=-0.024 *p* = 0.676	*r*=-0.077 *p* = 0.188	*r* = 0.021 *p* = 0.737	*r*=-0.022, *p* = 0.725	*r*=-0.003 *p* = 0.96	*r*=-0.123* *p* = 0.034	*r* = 0.001, *p* = 0.991
Perifovea Inferior SCP VD (%)	*r*=-0.081 *p* = 0.169	*r* = 0.019 *p* = 0.739	*r*=-0.04 *p* = 0.491	*r* = 0.019 *p* = 0.753	*r*=-0.041, *p* = 0.502	*r*=-0.025 *p* = 0.687	*r*=-0.109 *p* = 0.061	*r* = 0.015, *p* = 0.787
Whole Image DCP VD (%)	*r*=-0.089 *p* = 0.131	*r*=-0.011 *p* = 0.853	*r*=-0.068 *p* = 0.245	*r*=-0.002 *p* = 0.97	*r*=-0.075, *p* = 0.224	*r*=-0.097 *p* = 0.113	*r*=-0.108 *p* = 0.064	*r*=-0.056, *p* = 0.326
Perifovea Temporal DCP VD (%)	*r*=-0.127* *p* = 0.03	*r*=-0.034 *p* = 0.566	*r*=-0.096 *p* = 0.1	*r* = 0.02 *p* = 0.74	*r*=-0.074, *p* = 0.232	*r*=-0.109 *p* = 0.076	*r*=-0.102 *p* = 0.08	*r*=-0.065, *p* = 0.253
Perifovea Superior DCP VD (%)	*r*=-0.08 *p* = 0.172	*r*=-0.005 *p* = 0.931	*r*=-0.046 *p* = 0.434	*r* = 0.054 *p* = 0.383	*r*=-0.025, *p* = 0.688	*r*=-0.059 *p* = 0.338	*r*=-0.086 *p* = 0.142	*r* = 0.01, *p* = 0.855
Perifovea Nasal DCP VD (%)	*r*=-0.029 *p* = 0.621	*r*=-0.01 *p* = 0.87	*r*=-0.044 *p* = 0.45	*r*=-0.015 *p* = 0.807	*r*=-0.063, *p* = 0.31	*r*=-0.078 *p* = 0.207	*r*=-0.098 *p* = 0.093	*r*=-0.011, *p* = 0.853
Perifovea Inferior DCP VD (%)	*r*=-0.1 *p* = 0.088	*r* = 0.005 *p* = 0.936	*r*=-0.048 *p* = 0.412	*r*=-0.081 *p* = 0.188	*r*=-0.106, *p* = 0.084	*r*=-0.122* *p* = 0.046	*r*=-0.056 *p* = 0.334	*r*=-0.096, *p* = 0.089

## Discussion

This cross-sectional study of 296 healthy subjects with OCTA imaging and SphygmoCor XCEL system data to evaluate the correlation between macular VD and arterial stiffness. We noted a sex-based difference in vascular density on 3 × 3 vs. 6 × 6 mm scans: male subjects had significantly higher PWV values. At the same time, female DCP VDs were, on average, higher except for foveal DCP VD on 3 × 3 mm scans. Foveal DCP VD was found to be higher in male subjects. On 6 × 6 mm OCTA scans, female participants had significantly higher parafoveal SCP VD values and larger FAZs, with no significant difference among other parameters. The 6 × 6 mm scans capture a larger macular area. The difference in vascular density on 3 × 3 vs. 6 × 6 mm scans could be explained by the larger FAZ size seen among women.

Our study found that women had higher VD values than men across most measurements. Statistically significant differences were observed in specific regions: in the 3 × 3 mm scans, women exhibited higher VD values in both the whole image and the parafoveal DCP. In the 6 × 6 mm scans, women had significantly higher VD values in the whole image and the perifoveal SCP. These results suggest a sex-related variation in retinal microvascular density, which may have implications for understanding sex-specific differences in retinal vascular health. A similar study by Shahlaee et al. on healthy subjects, men and women, showed that the mean DCP VD was generally significantly higher than the SCP VD. Specifically, the DCP VD was found to be 52 ± 2.4%, while the SCP VD was only 46 ± 2.2%. Interestingly, their study did not report a sex-based difference in VD values [29]. According to a study by You et al., lower SCP VD maintained a significant association with male sex in multivariate analysis [30]. Richter et al. also reported that the male gender was associated with reduced perifoveal and macular VD [31]. However, other correlative studies have found no associations between macular VD and sex [32, 33]. In a study on healthy participants by Heidarzadeh et al., they reported the normative values for central retina VDs, and whole image SCP and DCP VDs were found to be 45.9 ± 2.6%, 50.2 ± 3%, respectively [34]. These values in our study were 46 ± 2.4% and 50.2 ± 2.9%. They also reported that these values were significantly higher in females and younger participants.

Measuring arterial stiffness is a valuable approach to predicting cardiovascular disease, and combining this metric with standard measurements improves accuracy in identifying high-risk individuals. High blood flow organs such as the brain and the eye are vulnerable to excessive pressure and flow fluctuations, resulting in gradual microvascular ischemia and tissue damage over time [5]. Several ocular and systemic diseases can impact macula VD, and certain ocular diseases can also affect PWV parameters. Penteado et al. found that the loss of macular VD is linked to visual field defects on Humphrey visual field 10 − 2 testing, and that monitoring macular VD can aid in the management of glaucoma patients [15]. Additionally, Hou et al. discovered that inter-eye VD asymmetry was significantly greater among glaucoma suspect patients than in healthy controls [19]. In another glaucoma study, it was determined that macular VD was reduced in such patients with primary open angle glaucoma compared to healthy individuals [18]. Lastly, Lee et al. concluded that outer macular VD was the most effective measurement for diagnosing glaucoma compared to other VD measurements. VD measurements were also a reliable marker of glaucoma diagnosis in patients with moderate to high myopia [21].

Moreover, in a case-control study on patients with pseudoexfoliation glaucoma, carotid-femoral PWV was found to be significantly higher [10]. Bourouki et al. found that PWV and AI values were higher in subjects with primary open-angle and pseudoexfoliation glaucoma than healthy subjects [12]. In contrast to VD values in these glaucoma studies, arterial stiffness has been found to not correlate with a diagnosis of glaucoma [11].

A study conducted on 15 healthy subjects and 22 patients with various retinal diseases found that the retinal diseases group had a lower macular SDP VD value compared to healthy controls. This suggests that individuals with retinal diseases have a reduced amount of blood flow to inner retinal layers [14]. Li et al. conducted a cross-sectional study on 97 diabetic patients and 85 healthy controls. They revealed that diabetic patients had a significantly lower retinal VD compared to healthy controls. The study suggested that OCTA is a valuable measure for early detection of diabetic retinopathy [20]. Ogawa et al. demonstrated that patients with diabetic retinopathy had a higher brachial-ankle PWV, but no significant differences were found in Aix [6]. Another study conducted on patients with branch retinal vein occlusion and hypertensive subjects showed that they had an impaired PWV compared to healthy adults [8]. Lastly, patients with age-related macular degeneration had an increased systemic arterial stiffness [9].

Similar to ocular pathologies, systemic diseases have also been found to impact macular VD values. A study found a significant decline in parafoveal VD in Alzheimer’s patients compared to healthy controls and suggested it as an early biomarker of the disease [16]. We came across two studies that examined the impact of COVID-19 on macula VD. Another study, which involved 27 children who had recovered from COVID-19, showed a significant increase in macula SCP VD compared to a group of 45 controls [17]. However, Abrishami et al. showed that the patients who had recovered from COVID-19 had a progressive decrease of VD at the follow-up visit 3 months after COVID-19 infection [22].

Previous case-control studies have investigated the relationship between macula VD and PWV in various eye conditions. However, our study is the first to explore this correlation in healthy individuals. The outcomes of our study, which employed the Pearson correlation coefficient, have indicated a negative correlation between the whole image DCP VD in 3 × 3 mm OCTA scans and both systolic and diastolic BPs. This suggests that hypertensive damage may occur mainly in the DCP of the retinal vasculature. Furthermore, we have found a negative correlation between PWV and parafoveal DCP VDs. However, no correlation has been established between the macula VDs and the aortic diastolic and systolic pressures.

These observations hold significant implications for the management of hypertension. The findings suggest that the DCP of the retinal vasculature may be particularly vulnerable to hypertensive damage. Therefore, monitoring DCP VD may be a valuable tool for assessing the severity of hypertensive damage in patients. Moreover, the observed negative correlation between PWV and parafoveal DCP VDs highlights the importance of monitoring PWV in patients with hypertension. As PWV is an established marker of arterial stiffness, the negative correlation with parafoveal DCP VDs suggests that arterial stiffness may contribute to retinal microvascular dysfunction.

Our study reveals that all parafoveal SCP and DCP VDs correlate negatively with age. Interestingly, the fovea SCP and DCP VDs had no statistically significant correlation with age. Intriguingly, we also found that AI was the only value that demonstrated a positive correlation with age, while all other FAZ parameters failed to exhibit any correlation. Moreover, our results show that age negatively correlates with SCP VD in the perifoveal region, whereas no correlation was detected between foveal VD and age. These findings suggest that age is a critical factor that needs to be considered when analyzing SCP and DCP VDs in the parafovea region. Also, You et al. reported that the lower VD was borderline associated with older age in multivariate analysis [30]. Fernández-Vigo et al. reported a negative correlation between macular VD and age, which is consistent with our findings [32]. In a separate investigation conducted by Pujari et al., it was observed that the VD values exhibited a declining trend across all macular regions from the second to the sixth decade [35].

Our study encountered certain limitations regarding enrolling participants younger than 20 and older than 60. Nevertheless, the substantial sample size permitted a comprehensive analysis. Additionally, while chronic smokers were not excluded from the study, this subgroup comprised only 14 individuals with a median of 1.77 pack-years.

## Conclusion

Our study revealed significant sex-based differences in macular vascular density and FAZ, with higher macular SCP and DCP VD values in all regions among female participants. This difference was statistically significant in the whole image and parafoveal sectors for DCP VDs in 3 × 3 mm scans and in the whole image and perifoveal sectors for SCP VDs in 6 × 6 mm scans. Females also exhibited significantly lower aortic pressures, PWV, and SphygmoCor reference age values in arterial stiffness measures, while men’s AIx values were notably higher. Additionally, older age negatively correlated with SCP and DCP VDs, except for foveal SCP and DCP VDs. Moreover, PWV demonstrated a significant negative correlation with parafoveal DCP VDs in 3 × 3 mm scans and whole image SCP VD in 6 × 6 mm scans.

## Electronic supplementary material

Below is the link to the electronic supplementary material.

Supplementary Material 1

## Data Availability

No datasets were generated or analysed during the current study.

## Abbreviations

AI: Acircularity index
AIx: Augmentation index
BMI: Body mass index
BP: Blood pressure
CBP: Central blood pressure
COVID-19: Corona virus disease 2019
DCP: Deep capillary plexus
FAZ: Foveal avascular zone
FD: Foveal vessel density
MUMS: Mashhad university of medical sciences
OCTA: Optical coherence tomography angiography
POCM: Persian organizational cohort study
PWV: Pulse wave velocity
SCP: Superficial capillary plexus
VD: Vessel density
